# Intuitive Risk Equation for Post-Transplant Bloodstream Infection Prediction: A Symbolic Regression Approach

**DOI:** 10.3390/biomedicines14040840

**Published:** 2026-04-07

**Authors:** Sungsu Oh, Jeongin Jang, Yunseong Ko, Hyunsu Lee, Seungjin Lim

**Affiliations:** 1 Department of Physiology, School of Medicine, Pusan National University, Yangsan-si 50612, Republic of Korea; oss1997082@pusan.ac.kr (S.O.); jeongin122201@gmail.com (J.J.); 2 School of Medicine, Pusan National University, Yangsan-si 50612, Republic of Korea; dbstjdrh@pusan.ac.kr; 3 Research Institute for Convergence of Biomedical Science and Technology, Pusan National University Yangsan Hospital, Yangsan-si 50612, Republic of Korea; 4 Medical Research Institute, School of Medicine, Pusan National University, Yangsan-si 50612, Republic of Korea; 5 Division of Infectious Diseases, Department of Internal Medicine, Pusan National University Yangsan Hospital, Yangsan-si 50612, Republic of Korea

**Keywords:** liver transplantation, postoperative bloodstream infection, survival analysis, machine learning, symbolic regression, disease prediction

## Abstract

**Background:** Liver transplant recipients are highly susceptible to infectious complications due to surgical invasiveness and immunosuppressive therapy, and post-transplant bloodstream infection is associated with substantial morbidity and mortality. Although several prediction models for bloodstream infection have been proposed, most focus on emergency department or general ward populations and rely on black-box approaches. This limits their applicability and clinical interpretability in liver transplant settings. Therefore, this study aimed to develop predictive models for post-transplant bloodstream infection using preoperative and perioperative clinical data and to derive an interpretable risk equation through symbolic regression. **Methods:** We conducted a retrospective observational study including 245 adult liver transplant recipients treated at a single tertiary center. Clinical and laboratory variables were extracted from electronic medical records and analyzed using standard statistical methods. For prediction tasks, multiple conventional machine learning models were developed and compared with a symbolic regression-based model. Predictive performance and model interpretability were evaluated using discrimination metrics and Shapley Additive Explanations. **Results:** Post-transplant bloodstream infection occurred in 82 patients (33.4%). In the test set, conventional machine learning models showed modest discriminative performance (area under the curve, 0.53–0.64). The symbolic regression model achieved comparable discrimination (area under the curve, 0.63) while providing transparent, threshold-based risk equations. While conventional models primarily relied on laboratory variables, symbolic regression additionally identified perioperative clinical factors and viral serologic markers as important predictors. **Discussion:** Although overall predictive performance was modest, symbolic regression highlighted viral serologic markers as potential indicators of immunologic vulnerability, extending beyond standard laboratory predictors. **Conclusions:** This interpretability-focused approach may inform future risk stratification models incorporating richer perioperative data.

## 1. Introduction

Globally, chronic liver disease and its complications account for approximately two million deaths annually [1]. Chronic liver disease is strongly associated with progressive liver injury, leading to hepatic fibrosis, cirrhosis, organ failure, or hepatocellular carcinoma. The major etiological factors include hepatitis B and C virus infections, alcoholic liver disease, and metabolic dysfunction-associated steatotic liver disease (MASLD).

Liver transplantation (LT) has become an essential therapeutic option for patients with end-stage liver disease, and the number of procedures has steadily increased worldwide, reaching 34,694 cases in 2021. This represents a 6.5% increase compared to 2020 and a 20% increase compared to 2015 [2]. Advances in surgical techniques and immunosuppressive therapy have significantly improved post-transplant survival compared to earlier eras [3]. However, many liver transplant recipients are already immunocompromised due to advanced liver disease, including cirrhosis, even before undergoing transplantation and immunosuppressive therapy [4]. Consequently, they are at high risk of infection, which remains the most common post-transplantation complication and a leading cause of morbidity and mortality [5].

This vulnerability is evident in epidemiological data. Reported infection rates within one month after LT range from 20% to 70%, with more than two-thirds of patients experiencing infection-related complications [6,7]. A retrospective analysis of 222 adult transplant recipients at a tertiary hospital in Korea showed that bloodstream infections (BSI) occurred in 28.8% of patients within one year, and these BSIs were closely associated with reduced overall survival [8]. Similarly, a recent multicenter retrospective study in China reported that bacterial infections occurred in 26.6% of recipients within two months after transplantation [9]. Therefore, while LT provides a critical survival opportunity for patients with end-stage liver disease, the combination of invasive procedures, immunosuppression, and patient-related vulnerabilities renders recipients highly susceptible to infections. These infections remain a major clinical concern.

Among various infections affecting liver transplant recipients, such as surgical site infections, pneumonia, and urinary tract infections, BSI is considered the most critical [4,10,11]. Post-transplant BSI can rapidly progress to sepsis and multiorgan failure if early diagnosis is delayed, serving as one of the major causes of graft failure and reduced overall survival [12]. Identifying high-risk patients for potential BSI in advance and implementing timely interventions are considered crucial for improving clinical outcomes. Capturing early warning signals of BSI and intervening before onset could optimize the timing of antibiotic administration. However, despite the importance of early detection, the nature of LT makes this difficult. In the immediate post-transplant period, the immunosuppressed state often masks typical infection symptoms, making early clinical recognition challenging [13]. Consequently, no clear criteria currently exist for predicting BSI in advance [14]. Although challenging, predicting and screening patients at high risk of developing BSI is essential, as it can improve patient prognosis, avoid unnecessary antibiotic use, and reduce the incidence of multidrug-resistant pathogens [4,11,14,15,16,17].

Recently, several predictive models using electronic medical records (EMR) and laboratory data have been developed to forecast infectious events; however, most of these models target emergency department or general patient populations rather than focusing on specific post-surgical cohorts such as liver transplant recipients [18,19,20,21]. Biomarkers such as procalcitonin have been investigated for BSI screening, but their performance in clinical practice remains inconsistent [22,23,24,25]. Blood culture is still considered the diagnostic gold standard, yet it is limited by false positives and false negatives, and pathogen growth typically requires several days, thereby delaying timely therapeutic intervention [26,27,28,29,30]. These limitations highlight the need for novel approaches tailored to liver transplant recipients. In this context, machine learning-based predictive models offer a promising strategy to identify patients at high risk of BSI, enabling earlier intervention and improved clinical outcomes.

In line with this need, machine learning has in recent years been increasingly applied in clinical research to analyze large-scale, high-dimensional datasets, enabling the identification of potential risk factors and improving predictive accuracy [15,31,32,33,34,35]. Infection prediction, which often involves multiple complex clinical variables, is a domain where the nonlinear pattern recognition capacity of machine learning models offers significant advantages [36]. For example, one study predicted sepsis in liver transplant recipients up to 12 h before onset with a high area under the curve (AUC) of 0.97 using continuously monitored vital signs such as heart rate and blood pressure [37]. Another study demonstrated that an extreme gradient boosting (XGBoost) model incorporating 14 clinical features achieved an AUC of 0.784 in predicting post-transplant pneumonia [15]. Moreover, interpretable models that can be applied in real time in clinical settings provide value not only in predictive accuracy but also in clinical acceptance [38]. By offering intuitive explanations for infection risk, such models can facilitate clinician decision-making and improve communication with patients who have limited health literacy.

The aim of this study is to develop a machine learning-based predictive model for the early detection of BSI in liver transplant recipients using preoperative clinical data and to derive an interpretable risk equation through symbolic regression (SR), which enables the generation of transparent and intuitive mathematical expressions [39,40,41]. In addition to model development, we further explored key predictive variables and proposed a biomarker discovery framework to identify clinically meaningful predictors. To the best of our knowledge, this is the first study to apply machine learning and SR to predict BSI in liver transplant recipients using preoperative and intraoperative data.

The major contributions of our study are as follows: (1) we employ SR to derive interpretable risk equations that support clinical decision-making; (2) we explore and analyze key predictive variables to identify clinically relevant and potentially novel biomarkers; and (3) we propose an interpretability-optimized symbolic regression-based framework for disease prediction, designed to balance predictive performance and clinical interpretability in EMR-based risk modeling.

## 2. Materials and Methods

This retrospective observational study included patients who underwent liver transplantation (LT) at a single center, Pusan National University Yangsan Hospital, between 2010 and 2023. A total of 245 adult patients (≥18 years) who received either living donor liver transplantation (LDLT) or deceased donor liver transplantation (DDLT) were included. Patients were excluded if they had insufficient EMR, died within 24 h after surgery, or underwent transplantation at another institution and were only followed at our center. The study was conducted with the approval of the Institutional Review Board (IRB) of Pusan National University Yangsan Hospital (IRB approval number: 55-2025-121). Clinical and laboratory data were collected through direct review of the EMR by researchers.

Regarding pre-transplant infections, patients with active infections underwent transplantation only after infection control was achieved. In some cases where residual infection remained, transplantation was performed at the discretion of the surgeon, with appropriate antibiotic therapy maintained as needed.

BSI was determined based on blood culture results obtained during the observation period. Blood cultures were processed using routine clinical microbiological procedures. Microorganisms isolated from positive blood cultures were identified using matrix-assisted laser desorption/ionization time-of-flight mass spectrometry (MALDI-TOF MS) and an automated identification system (VITEK 2, bioMérieux, Marcy-l’Étoile, France). Blood cultures were collected from peripheral veins, central venous lines, or arterial lines, and all results were integrated to identify organisms and determine species-level information. BSI was defined according to established diagnostic criteria from previous studies [14,20,42]. The presence of a non-contaminant pathogenic organism in at least one blood culture was considered indicative of BSI. Organisms considered as common contaminants are listed in Table A6. In cases where organisms commonly regarded as contaminants were isolated, BSI was defined only if the same organism was identified in additional blood cultures obtained within 24 h. Fungal isolates identified from blood cultures were also included as causative pathogens and analyzed within the same BSI framework. This study focused on the occurrence of BSI after liver transplantation based on predefined microbiological criteria. Source attribution of each BSI episode, including possible linkage to a previous chronic infection, was not specifically assessed.

### 2.1. Data Characteristics

The dataset used in this study comprised preoperative clinical information from liver transplant recipients, serving as input variables for both the development of machine learning-based predictive models and the derivation of an interpretable risk equation. In total, 83 variables were collected and classified into seven categories.

First, demographic information included sex, age, weight, and height, which were incorporated as predictors alongside other clinical variables.

Second, medical history data captured comorbidities and past medical conditions, including hypertension (HTN), diabetes mellitus (DM), cardiovascular disease, cerebrovascular disease, chronic lung disease (CLD such as COPD or asthma), chronic kidney disease (CKD), hepatic encephalopathy (HE), and ascites. Additionally, liver disease etiologies related to transplantation indications (e.g., alcoholic liver disease, chronic hepatitis B or C, autoimmune hepatitis), hepatocellular carcinoma (HCC) status, history of abdominal surgery with major surgical details, and portal vein thrombosis were included as structured variables.

Third, preoperative clinical status data encompassed variables reflecting patient severity and treatment requirements at admission or before surgery. These included level of consciousness (LOC), type of hospital ward at admission, need for preoperative mechanical ventilation, and use of continuous renal replacement therapy (CRRT).

Fourth, laboratory test data comprised complete blood count (CBC; white blood cell (WBC), platelet (PLT), etc.), liver function tests [aspartate aminotransferase (AST), alanine aminotransferase (ALT), and total bilirubin (TB)], renal function tests [creatinine (Cr) and blood urea nitrogen (BUN)], electrolytes, C-reactive protein (CRP), and coagulation markers such as prothrombin time–international normalized ratio (PT-INR). These laboratory parameters were repeatedly measured at multiple time points before and after surgery, but only admission and preoperative values were used for model development.

Fifth, infectious disease data included results of microbiological culture tests, molecular assays such as polymerase chain reaction (PCR), and serological evaluations using antigen and antibody tests. These examinations covered common bacterial and viral pathogens relevant to transplant recipients. Test results obtained during the preoperative period were documented either as qualitative outcomes (positive or negative) or as quantitative measurements.

Lastly, surgery-related data reflected both transplant procedure characteristics and intraoperative patient status. Variables included liver transplant type (LT type), retransplantation status, intraoperative minimum and maximum body temperature, and transfusion volumes of red blood cells (RBC), PLT, and fresh frozen plasma (FFP), quantified in units or pints. These variables served as indirect indicators of surgical stress, bleeding severity, and procedural complexity.

Importantly, the selection of clinical variables was guided not only by established standards such as the United Network for Organ Sharing (UNOS) criteria [43] but also by clinical insights from transplant infectious disease specialists, ensuring both clinical relevance and methodological rigor.

### 2.2. Preprocessing

Feature selection and preprocessing were performed to refine raw EMR-based data into a structured form suitable for machine learning analysis. The steps included removal of low-variance and high-missing-rate variables, imputation of missing values, transformation of infectious disease diagnostic data, variable encoding for SR, and standardization for linear models such as logistic regression (LR) and support vector machines (SVM).

Variables with minimal variance across samples, unlikely to contribute meaningfully to prediction, were removed using the VarianceThreshold method [44]. To ensure data quality, variables with more than 50% missingness were excluded from the analysis [45]. Most of the removed variables exhibited missing rates exceeding 90%, and only eight variables had missing rates between 50% and 90%. The remaining variables had a missing rate of 3.04%, which was considered manageable with simple imputation methods [46]. Normality was assessed using the Shapiro–Wilk test; variables meeting normality assumptions were imputed with the mean, whereas those not meeting assumptions were imputed with the median. For categorical variables, the mode was applied for imputation. For patient-specific variables with minimal temporal variability (e.g., height, weight), simple imputation was deemed insufficient; therefore, mean imputation was supplemented with an additional binary indicator variable denoting the presence of missingness. This approach incorporates missingness itself as a potentially informative clinical signal [47].

For infectious disease diagnostic data, categorical values were originally recorded in formats such as Positive (n), Gray Positive (n), Gray Negative (n), Negative (n), ND, and NaN, where *n* represented a corresponding numeric measurement (e.g., titer or count), or in clipped numeric formats such as >n or <n. Negative or untested results were treated as absence of pathogens or clinical determination of no infection. Positive (n) and Gray Positive (n) values were converted to the corresponding *n*, while Gray Negative (n), Negative (n), and ND were uniformly converted to 0. Entries without explicit counts (e.g., Positive, Positive Gray, Negative Gray, Negative) were recorded into a single categorical variable with four classes. Values reported in clipped numeric form such as >n or <n were transformed to *n* for model input.

**Figure 1:**
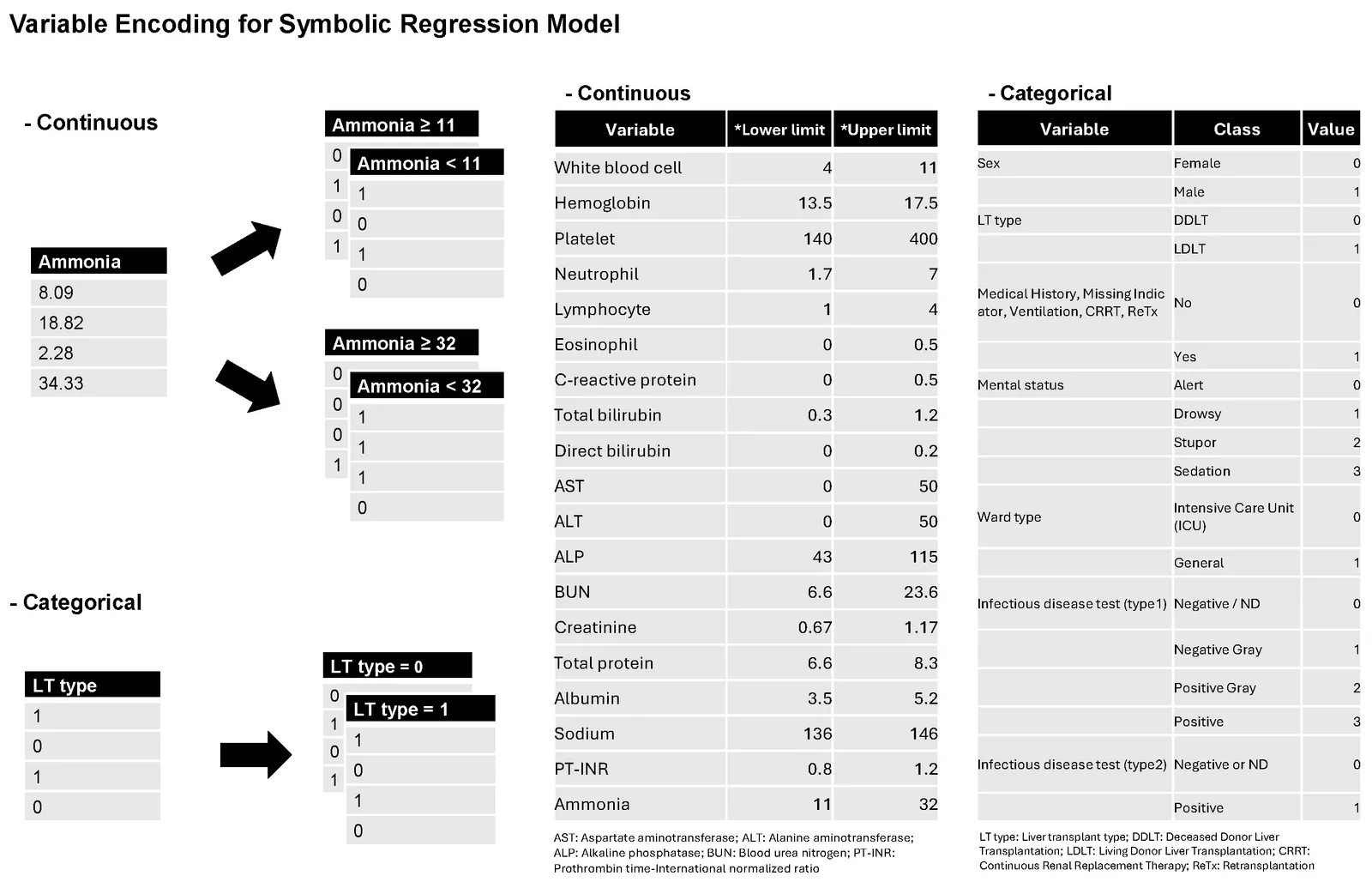
Variable encoding methods and representative examples. The **left** panel shows the binary encoding of continuous variables based on clinically defined normal reference ranges used at Pusan National University Yangsan Hospital and dummy encoding of categorical variables. The **right** panel presents representative threshold boundaries and corresponding encoded values. The arrows indicate the preprocessing steps of variables, showing the transformation from raw data to processed inputs. The asterisks (Upper limit, Lower limit) denote the upper and lower bounds of the clinical reference ranges, respectively.

To reduce model bias arising from differences in scale and range, continuous variables were standardized using z-score normalization for LR and SVM. Candidate features were finalized through iterative performance validation across multiple machine learning models and clinical expert review to ensure medical relevance. In contrast, for the symbolic regression (SR) model, normalization was not applied. Instead, continuous variables were binarized into logical variables (≥, <) based on clinically defined normal reference ranges routinely used at Pusan National University Yangsan Hospital. Categorical variables were likewise transformed into binary dummy variables (Figure 1).

In the full dataset, the ratio of post-transplant BSI to non-BSI cases was 82:163. To preserve this distribution, a stratified random split was applied, dividing the data into a training set (80%) and a test set (20%). For both traditional machine learning model development and SR model development, 5-fold cross-validation was used to optimize hyperparameters and generalization.

### 2.3. Model Development

Following the previous study that compared the performance of ML and SR methods for regression tasks [48], ML models were tuned using Halving Grid Search with 5-fold cross-validation, whereas SR models were optimized by exploring six predefined hyperparameter combinations, with constraints on training time or the number of evaluations. In the present study, ML models were tuned using Grid Search with 5-fold cross-validation, and SR models were optimized through a Gaussian process-based Bayesian optimization framework. Unlike the previous study, no restrictions on training time or evaluation budget were applied, as the objective was to evaluate predictive effectiveness rather than computational efficiency. Both models were trained until convergence, with early stopping incorporated into SR to determine convergence.

#### 2.3.1. Conventional Machine Learning Models

To predict post-transplant BSI, multiple conventional machine learning algorithms were employed, including L1-regularized LR (LR-L1), random forest (RF), SVM and XGBoost. Hyperparameter tuning was performed via grid search with 5-fold cross-validation. The classification threshold was determined using Youden’s J index, calculated from out-of-fold (OOF) predictions during the cross-validation process. These conventional models served as baseline comparators for evaluating the performance of the SR-based approach.

#### 2.3.2. Symbolic Regression Model

In addition to conventional algorithms, the Symscore model, an SR approach based on genetic algorithms, was applied to derive interpretable risk equations [49,50]. SR model calculates the risk score *R* as the sum of multiple binary conditions *S*, and the prediction $\hat{y}$ is obtained by applying the sigmoid function σ . The formulation is given as follows:

(1) $$\begin{array}{cc} R & =\sum_{j\in S}w_{j}x_{j}+b, \end{array}$$

(2) $$\begin{array}{cc} \hat{y} & =\sigma \left(R\right)=\frac{1}{1+e^{-R}} \end{array}$$

where $x_{j}\in \left[0,1\right]$ , $w_{j}$ , *b*, and *S* denote a binary variable indicating whether the condition is satisfied, the coefficient of the variable, the constant term, and the set of selected variables, respectively.

The symbolic regression (SR) model simultaneously learns both model structure and parameters by automatically searching for mathematical expressions from data. In this study, to ensure interpretability, the model was constrained such that only the addition operator (‘+’) was allowed in the resulting expressions. SR typically employs multi-objective optimization, balancing goodness of fit with interpretability or parsimony [39,41]. After variable encoding, approximately 200 candidate variables were explored, with log-loss prioritized as the primary objective function while allowing relatively flexible formula complexity.

For hyperparameter optimization, the same fitting procedure as the main training was applied, but with a reduced search space for candidate expressions. Hyperparameter optimization was conducted using Gaussian process-based Bayesian optimization [50,51]. The target parameters were three mutation probabilities—hoist mutation probability $p_{h}$ , point mutation probability $p_{m}$ , and subtree mutation probability $p_{s}$ —with their search ranges restricted between 0.0 and 0.1. A total of 20 optimization runs were performed, and the best-performing parameter set was applied to the final model training. For the SR model, OOF predictions from cross-validation were also used to calculate Youden’s index. The final model was selected as the equation with the lowest log-loss value, ensuring optimal predictive performance.

To evaluate model efficiency and identify the most distinct variables, we performed a sequential reduction analysis. Variables with lower scores were iteratively removed from the initial set. At each reduction step, the predictive performance was assessed by calculating the AUC on validation to determine the optimal cut-off point.

The machine learning models were implemented using Python (version 3.10.18) with scikit-learn (version 1.1.3), including RandomForestClassifier, LogisticRegression, SVC, StandardScaler, VarianceThreshold, StratifiedKFold, and GridSearchCV. The XGBoost model was implemented using the XGBoost library (version 3.0.5). Symbolic regression modeling was performed using gplearn (version 0.4.2), and Bayesian optimization was conducted using scikit-optimize (version 0.9.0).

### 2.4. Model Evaluation

Model performance was assessed using Receiver Operating Characteristic–Area Under the Curve (ROC-AUC), accuracy, recall, and F1 score. In addition, interpretability and feature contribution were assessed using Shapley Additive Explanations (SHAP) to visualize the contribution of key predictive features [52].

The AUC and its 95% confidence interval (CI) were estimated using the DeLong method [53], a nonparametric approach for evaluating the variance of AUC. The empirical AUC is defined as:

(3) $$\hat{AUC}=\frac{1}{mn}\sum_{i=1}^{m}\sum_{j=1}^{n}\phi \left(X_{i},Y_{j}\right)$$

where $X_{i}$ and $Y_{j}$ denote the prediction scores for positive and negative samples, respectively, and *m* and *n* represent the number of positive and negative samples. The indicator function ϕ(·) is defined as:

(4) $$\phi \left(X_{i},Y_{j}\right)=\left\{\begin{array}{cc} 1, & X_{i}>Y_{j}\\ 0.5, & X_{i}=Y_{j}\\ 0, & X_{i}<Y_{j} \end{array}\right.$$

The variance of $\hat{AUC}$ was estimated using DeLong’s nonparametric covariance approach, and 95% CIs were calculated accordingly. CIs for accuracy, recall, and F1-score were estimated using bootstrap resampling.

Decision curve analysis was performed to evaluate the clinical utility of the models. The net benefit was calculated as:

(5) $$\mathrm{Net}\;\mathrm{Benefit}\left(p_{t}\right)=\frac{TP}{N}-\frac{FP}{N}\cdot \frac{p_{t}}{1-p_{t}}$$

where TP and FP denote the numbers of true positives and false positives, respectively, *N* is the total number of patients, and $p_{t}$ represents the threshold probability. Model performance was compared with the “Treat All” and “Treat None” strategies.

## 3. Result

### 3.1. Baseline Characteristics and Time-to-BSI Analysis

A total of 245 liver transplant recipients were included in this study. Quantitative variable analysis was performed using the *t*-test, while categorical variables were analyzed using the chi-square test. With a significance threshold of p<0.05 , 18 of 83 variables were identified as significantly different between the BSI and non-BSI groups.

Analysis of demographic and medical history variables showed no statistically significant differences. Age, weight, and height were comparable across groups, and sex distribution did not differ significantly. Similarly, missing data indicators for height and weight were not associated with BSI occurrence. Regarding comorbidities, the prevalence of CLD, HTN, DM, HCC, HE, and CKD did not differ between the two groups (all *p* > 0.05). These results suggest that baseline demographic and medical history factors were not significant discriminators of BSI risk in this cohort (Table 1 and Table A1).

Analysis of clinical status and laboratory findings revealed several variables with significant differences. In the clinical domain, LOC (preoperative) was poorer in the BSI group, showing a lower proportion of alert patients. In the BSI group, a higher proportion of patients were admitted to or remained in the ICU during the preoperative period, and both mechanical ventilation and CRRT were significantly more common preoperatively (not significant at admission) (Table 1 and Table A2).

Among laboratory tests, patients with BSI showed lower lymphocyte counts (admission and preoperative) and higher neutrophil counts (preoperative). Significant elevations were also observed in TB (admission and preoperative), BUN (admission), Cr (admission), PT-INR (preoperative), and ammonia (preoperative). Electrolyte imbalance was notable, with sodium levels at both admission and preoperative stages being significantly lower in the BSI group. Additionally, WBC count (preoperative) was significantly higher in the BSI group. These results highlight that inflammatory markers, hepatic and renal function parameters, electrolyte status, and preoperative clinical condition were closely linked to the risk of post-transplant BSI (Table A3).

Analysis of infectious disease test results demonstrated no statistically significant differences between patient groups. Preoperative viral markers and serologic markers, including tests for cytomegalovirus (CMV), Epstein–Barr virus (EBV), hepatitis B and C viruses (HBV, HCV), and human immunodeficiency virus (HIV), showed comparable distributions across groups (all p>0.05 ). These findings suggest that baseline viral and serologic profiles were not significant discriminators of post-transplant BSI risk in this cohort (Table A4).

Analysis of surgical variables identified several factors with significant differences. Intraoperative temperature parameters (minimum and maximum) did not differ between groups. However, patients who developed BSI required significantly higher amounts of RBC transfusion (*p* = 0.005) and FFP transfusion (*p* = 0.009). Additionally, LT type (DDLT) was significantly associated with BSI occurrence (*p* = 0.021). PLT transfusion and retransplantation status (ReTx) showed no significant differences. These results suggest that transfusion burden and transplant type were important perioperative factors linked to post-transplant BSI risk (Table A5).

Among the 245 liver transplant recipients, BSI occurred in 33.4%, underscoring its role as a major and common complication following transplantation. Causative pathogens were defined as organisms detected in blood cultures that were not considered common contaminants, or as common contaminants identified in repeated blood cultures within 24 h. The counting of causative pathogens was based on those detected in samples meeting the diagnostic criteria. If multiple causative pathogens were detected in a sample, each pathogen was counted separately. Analysis of causative pathogens showed that Gram-positive bacteria were most frequently identified, while Gram-negative bacteria and fungi were also detected at notable proportions (Table 2). This distribution indicates that BSI in liver transplant patients is not confined to a single microbial group, and that fungal infections should also be regarded as significant risk factors in immunosuppressed states.

**Figure 2:**
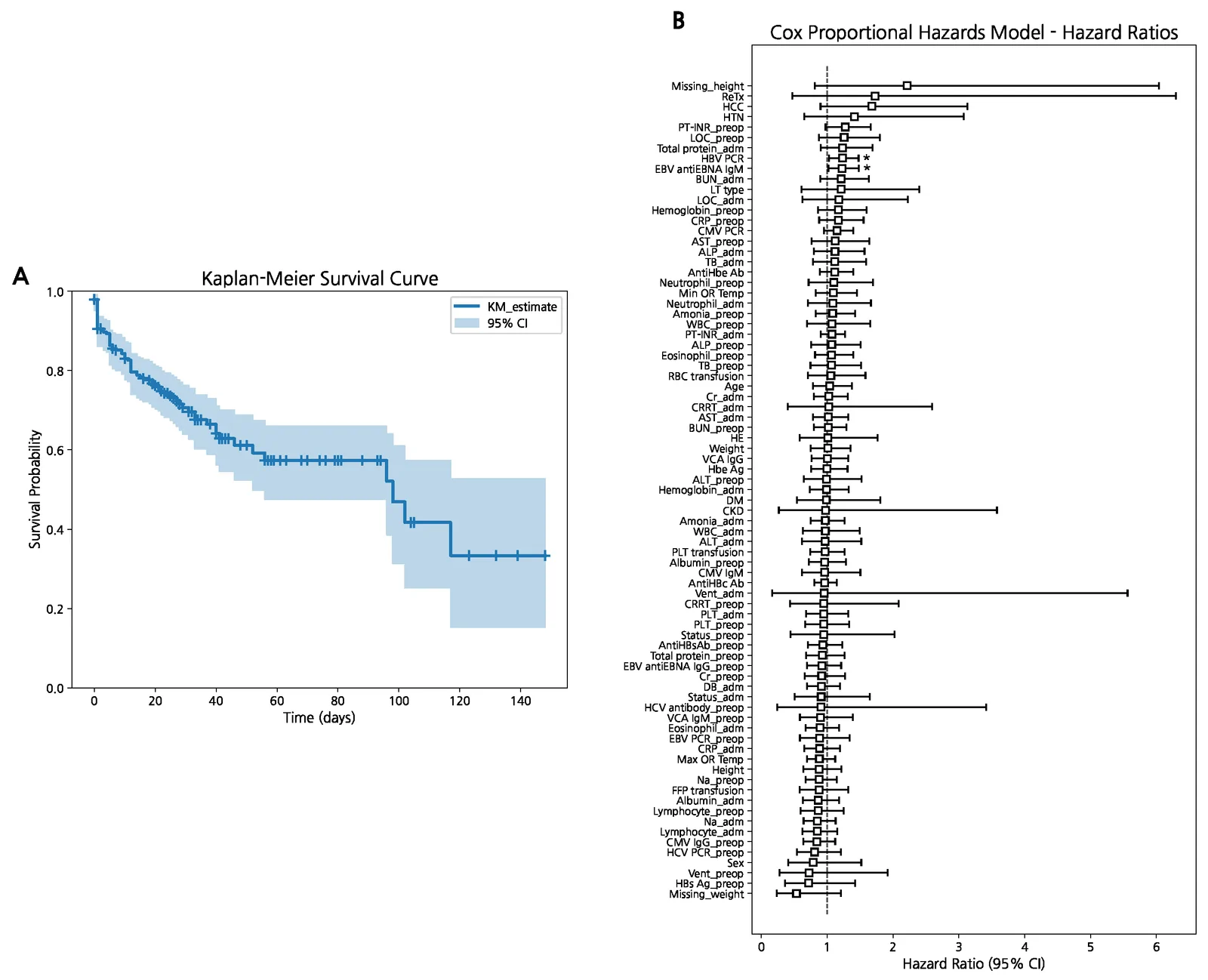
(**A**) Kaplan–Meier survival curve for BSI occurrence, showing the cumulative probability of remaining free from BSI over time. A steep decline is observed in the early postoperative period, with a further decrease around 100 days, though interpretation is limited by wide CIs. (**B**) Forest plot of hazard ratios from the Cox proportional hazards model, where hazard ratios greater than 1 indicate increased risk of post-transplant BSI; significant associations are observed for HBV PCR and EBV anti-EBNA IgM. An asterisk (*) indicates variables with a *p*-value < 0.05.

Figure 2A illustrates the Kaplan–Meier survival curve for BSI occurrence in liver transplant recipients [54]. Survival probabilities were estimated with censoring accounted for, and the shaded areas around the curve indicate 95% CIs. A marked decline was observed within the first 30 days, after which the slope flattened. At longer follow-up, survival probability dropped below 0.5, though the widening CIs reduced the precision of these estimates due to smaller sample sizes. These time-dependent risk patterns identified through Kaplan–Meier analysis reinforce the clinical need for early prediction and intensive monitoring of BSI after LT.

To identify time-dependent risk factors for BSI, multivariable Cox proportional hazards regression analysis was performed. Two preoperative viral markers, EBV anti-EBNA IgM and HBV PCR, were significantly associated with BSI occurrence ( p<0.05 ). Their hazard ratios were 1.228 (SE = 0.096) and 1.232 (SE = 0.093), respectively, indicating that both variables contributed to increased risk. Figure 2B presents the hazard ratios (HR) and 95% CIs in a forest plot. Only EBV anti-EBNA IgM and HBV PCR showed HRs exceeding 1 with statistical significance, while no other variables demonstrated meaningful differences in risk.

### 3.2. Model Performance Evaluation

**Figure 3:**
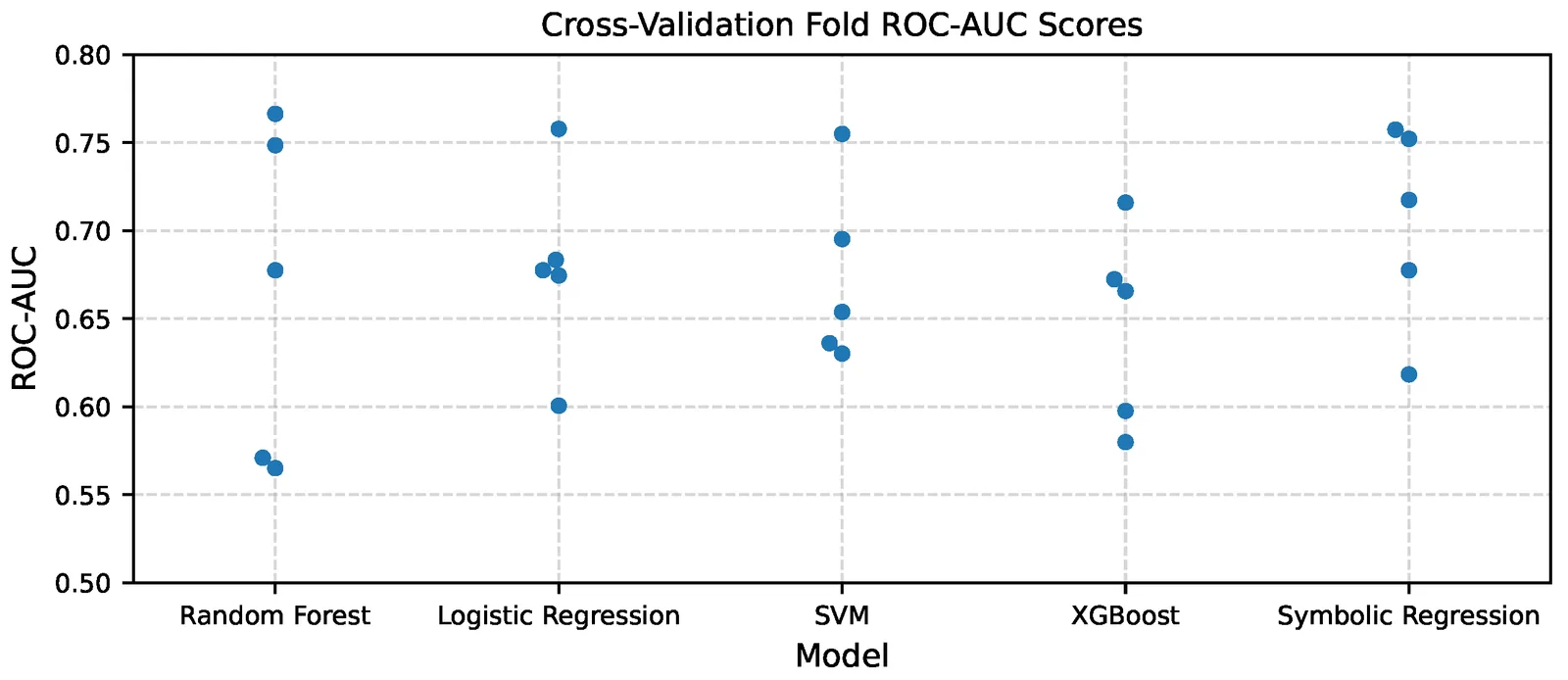
Cross-validation ROC-AUC comparison showing comparable performance across models, with relatively stable results across folds and higher variability observed for the random forest model. Blue dots represent the ROC-AUC values obtained from each cross-validation fold.

**Figure 4:**
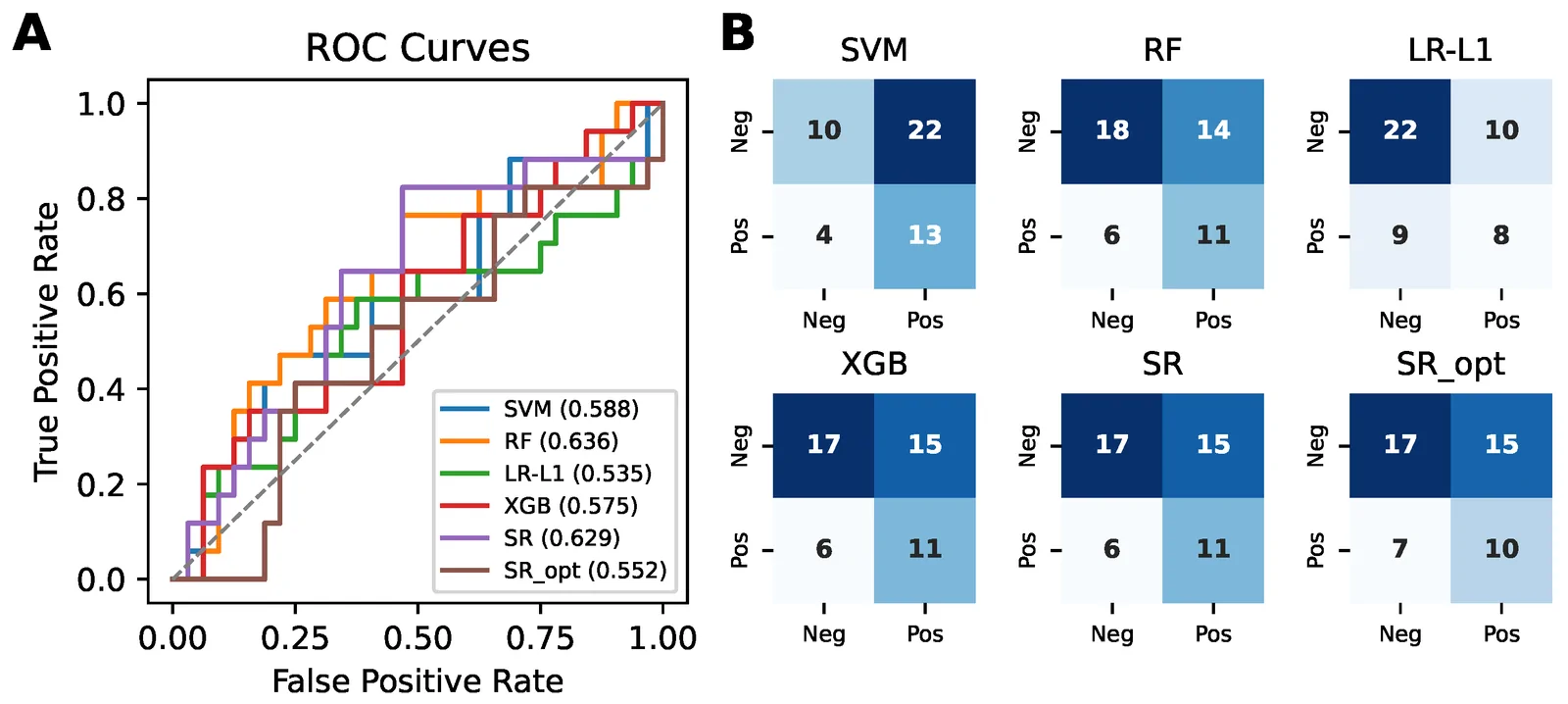
(**A**) ROC curves of five classifiers on test set. The dashed line represents the line of no-discrimination. Overall, none of the models demonstrated clearly superior classification performance. (**B**) Confusion matrices of each model. The blue color intensity reflects the magnitude of values in each cell, with darker shades indicating higher values. The highest AUC and F1-score were observed with RF, LR achieved the highest accuracy, and SVM showed the highest recall.

Building on the descriptive and survival analyses of post-transplant BSI presented in the previous section, we next evaluated predictive modeling approaches to assess the feasibility of early BSI prediction after LT. In this subsection, we compare the predictive performance of multiple algorithms, including traditional machine learning models and the SR model, and the interpretability-optimized SR model ( $SR_{opt}$ , detailed in Section 3.4). Model performance was compared using the liver transplant patient dataset, with results summarized in Figure 3 and Figure 4, and Table 3. In the cross-validation process (Figure 3), the traditional models and the SR model demonstrated the following ROC-AUC scores (mean ± SD): RF = 0.6657 ± 0.0952, LR = 0.6788 ± 0.0557, SVM = 0.6741 ± 0.0519, XGBoost = 0.6463 ± 0.0563, and SR = 0.7046 ± 0.0578, except for the RF model, which showed slightly higher variability, indicating comparable performance levels and relatively stable results across folds. Meanwhile, on the test set (Figure 4 and Table 3), models demonstrated AUC values from 0.53 to 0.63, indicating modest discriminative ability overall. Among these, RF achieved the high AUC (0.6360) and F1-score (0.5238), suggesting relative strength in identifying BSI cases. LR showed the highest accuracy (0.6122), while SVM demonstrated the highest recall (0.7647). The SR model achieved an AUC of 0.6287, comparable to traditional models, and also showed reasonable performance in recall (0.6471) and F1-score (0.5116). However, $SR_{opt}$ showed lower performance, with an AUC of 0.5524 and lower recall (0.5882) and F1-score (0.4762) in test set.

**Figure 5:**
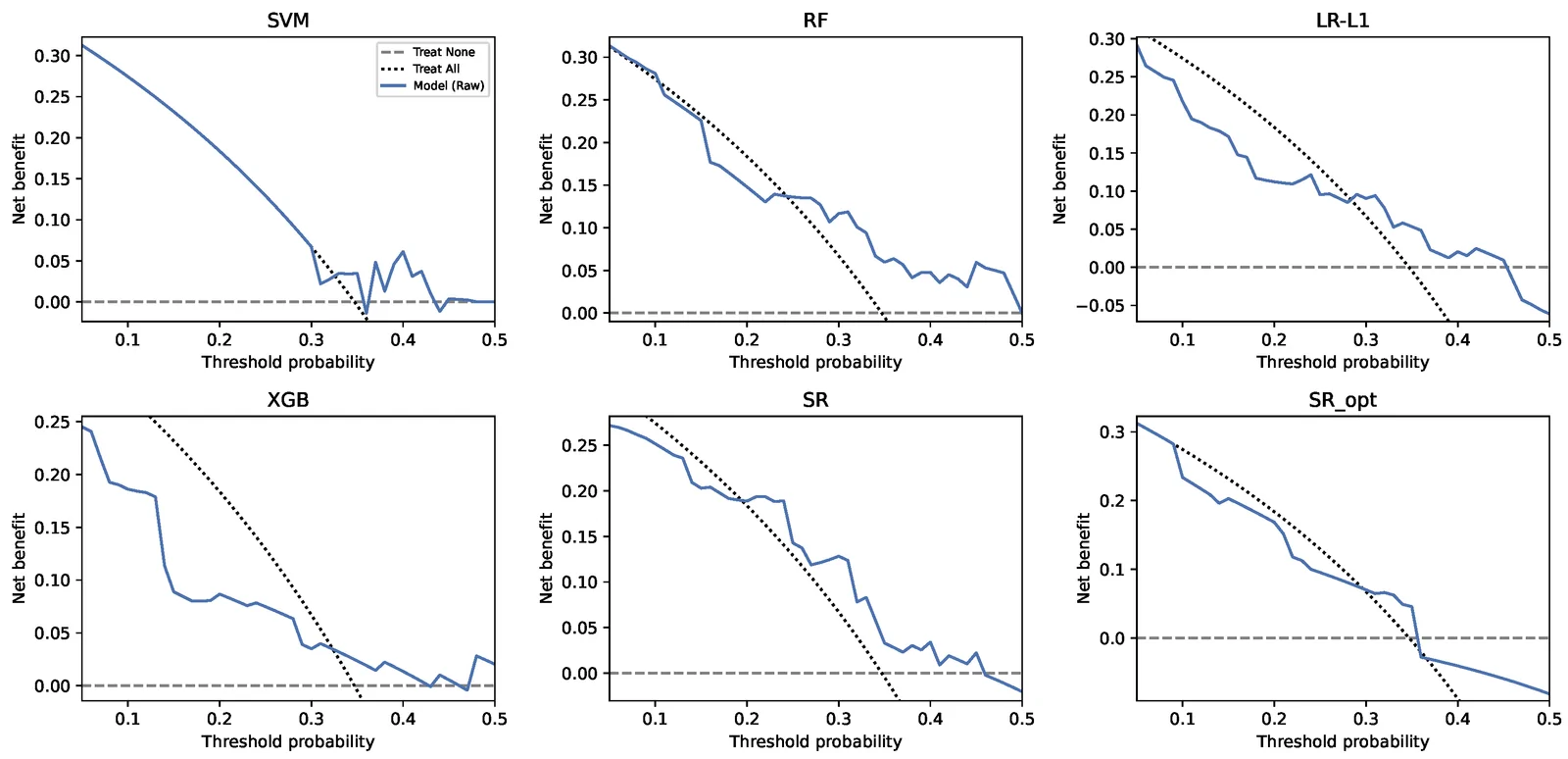
Decision curve analysis of predictive models for post-transplant bloodstream infection. Decision curve analysis shows the net benefit of each model across threshold probabilities. The solid blue line represents the net benefit of each predictive model, the dotted diagonal line indicates the “Treat all” strategy, and the horizontal dashed line represents the “Treat none” strategy. RF and SR models demonstrate relatively higher net benefit over most threshold ranges, while performance decreases at higher thresholds.

Decision curve analysis (Figure 5) was performed to evaluate the clinical utility of the models across different threshold probabilities. Net benefit reflects the trade-off between true positives and false positives in a decision-making context.

Most models showed higher net benefit than the “Treat None” strategy across a range of threshold probabilities; however, in several regions, their performance remained lower than that of the “Treat All” strategy. Notably, the RF and SR models demonstrated threshold ranges in which their net benefit exceeded both “Treat None” and “Treat All,” suggesting relatively improved decision performance within those intervals. However, at higher threshold probabilities, the net benefit of all models decreased, indicating limited clinical utility when stricter decision thresholds are applied.

### 3.3. Model Interpretation Using SHAP

**Figure 6:**
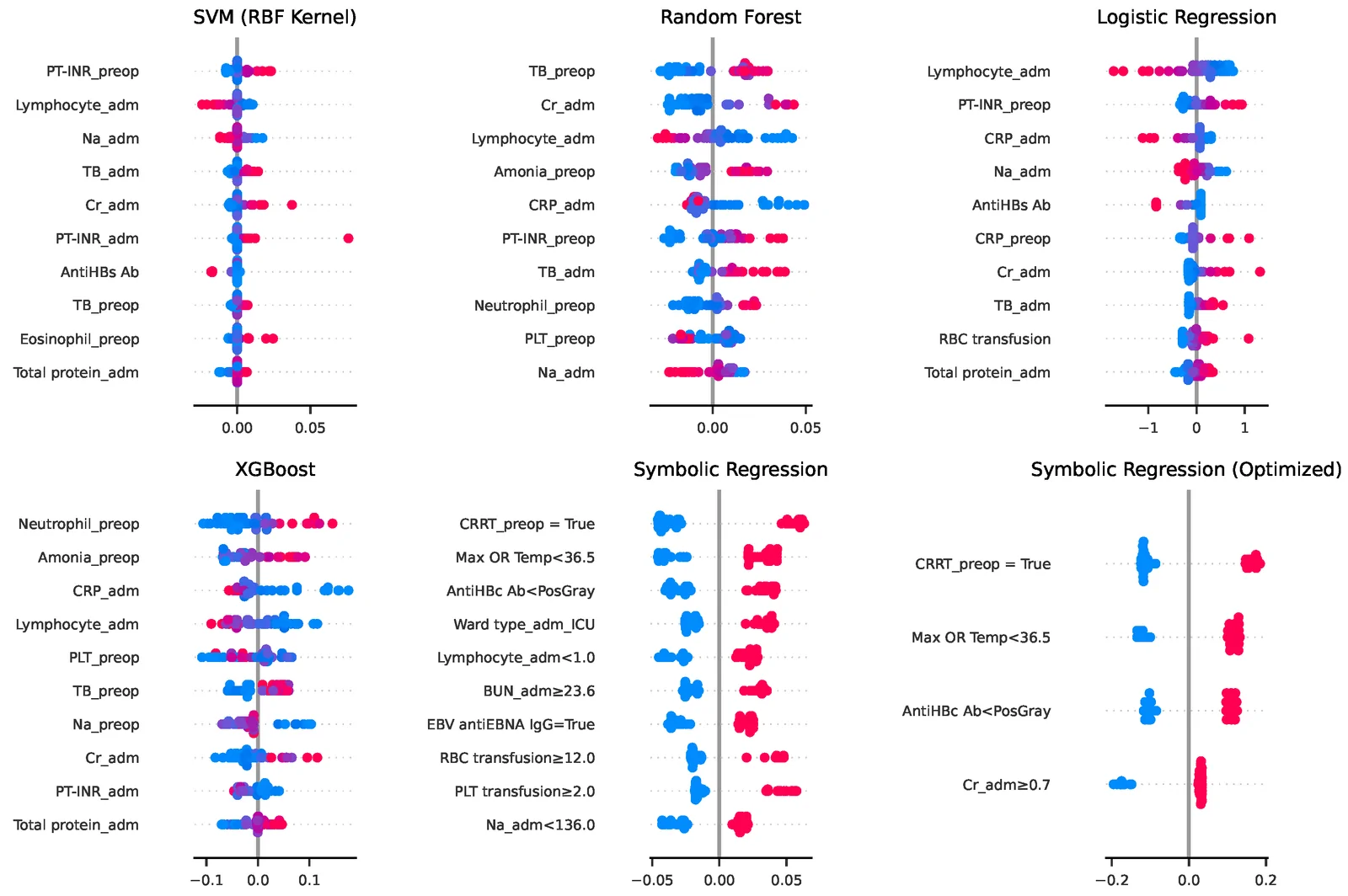
SHAP analysis illustrating the contribution of predictive variables across multiple models. Red dots indicate higher values of the corresponding variable, while blue dots indicate lower values. Compared with other machine learning models, the separation is most pronounced in the symbolic regression model.

To further investigate the clinical relevance of the predictive models, we performed feature attribution analysis using SHAP values. This subsection focuses on identifying key variables contributing to BSI prediction and comparing feature importance patterns across different algorithms. SHAP-based interpretability enabled consistent evaluation of predictive contributors across models, providing insights into how preoperative and perioperative clinical factors influence the modeled BSI risk of post-transplant BSI (Figure 6).

For the SVM (RBF kernel) model, PT-INR (preoperative), lymphocyte count (admission), Na (admission), Cr (admission), and TB (admission) were identified as key contributors. Additionally, viral serologic markers such as anti-HBs Ab contributed to model outputs. In the RF model, the most influential predictors included TB (admission, preoperative), Cr (admission), lymphocyte count (admission), ammonia (preoperative), and CRP (admission). In the L1-LR model, lymphocyte count (admission), PT-INR (preoperative), CRP (admission, preoperative), and Na (admission) emerged as the primary explanatory features. The XGBoost model highlighted neutrophil count (preoperative), ammonia (preoperative), CRP (admission), lymphocyte count (admission), and PLT count (preoperative) as the strongest predictors.

Across these four conventional ML models, several variables consistently appeared as important predictors, including lymphocyte count (admission), Na (admission), TB (admission, preoperative), and Cr (admission).

In contrast, the SR model expressed variable influence through interpretable threshold-based rules. Key contributors included CRRT use before surgery (CRRT_preop = True), ICU admission status (Ward type_adm_ICU), lymphocyte count at admission <1.0, BUN at admission ≥23.6, and sodium <136.0. Transfusion burden was also reflected through rules such as RBC transfusion ≥12 units and platelet transfusion ≥2 units. Intraoperative hypothermia (Max OR Temp <36.5 °C) also emerged as a relevant factor. For categorical variables such as anti-HBc Ab (0 = Negative, 1 = Negative Gray, 2 = Positive Gray, 3 = Positive), the rule anti-HBc Ab < PosGray indicated that non-positive results (values <2) contributed to the prediction.

Overall, lymphocyte count (admission) consistently contributed across all ML models. However, the SR model complemented these finding by highlighting additional predictors that were less prominent in conventional ML models—such as CRRT (preoperative), intraoperative temperature, ward type at admission, and specific serologic markers including anti-HBc Ab and anti-EBNA IgG. Together, these results suggest that while conventional ML models prioritize broadly informative laboratory indices, SR can reveal clinically relevant threshold-based conditions that may be overlooked by standard algorithms.

### 3.4. Explore SR Model

Building on the complementary insights described in the previous section, we further examined how the threshold-based rules generated by the SR model translate into an interpretable risk-scoring system. This subsection outlines the structure of the SR-derived equations, the relative contributions of their constituent rules, and how these components collectively form an interpretable and clinically applicable decision-making framework.

**Figure 7:**
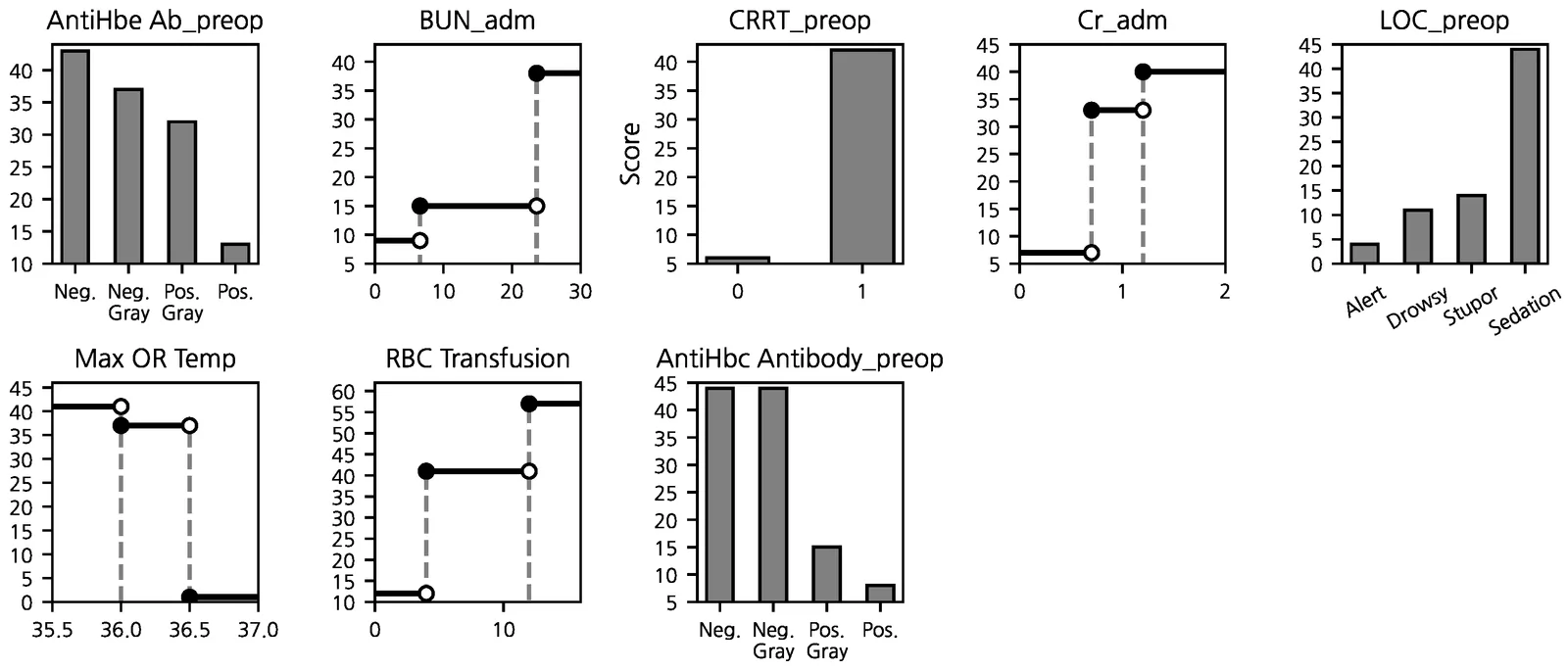
Range and score of the variables of top 10 term. Lines indicate value ranges, where filled (black) dots represent inclusive bounds and open (white) dots represent exclusive bounds.

Table 4 summarizes the top 10 variables with the highest coefficients ( $w_{j}$ ) along with their corresponding conditions and scores. These variables represent conditions that substantially contribute to increased risk when satisfied. When visualized using bar plots and cut-point plots, the score distribution clearly demonstrates the risk-enhancing ranges for each variable. This provides interpretability of the model and highlights its potential applicability in real-world clinical practice (Figure 7).

**Figure 8:**
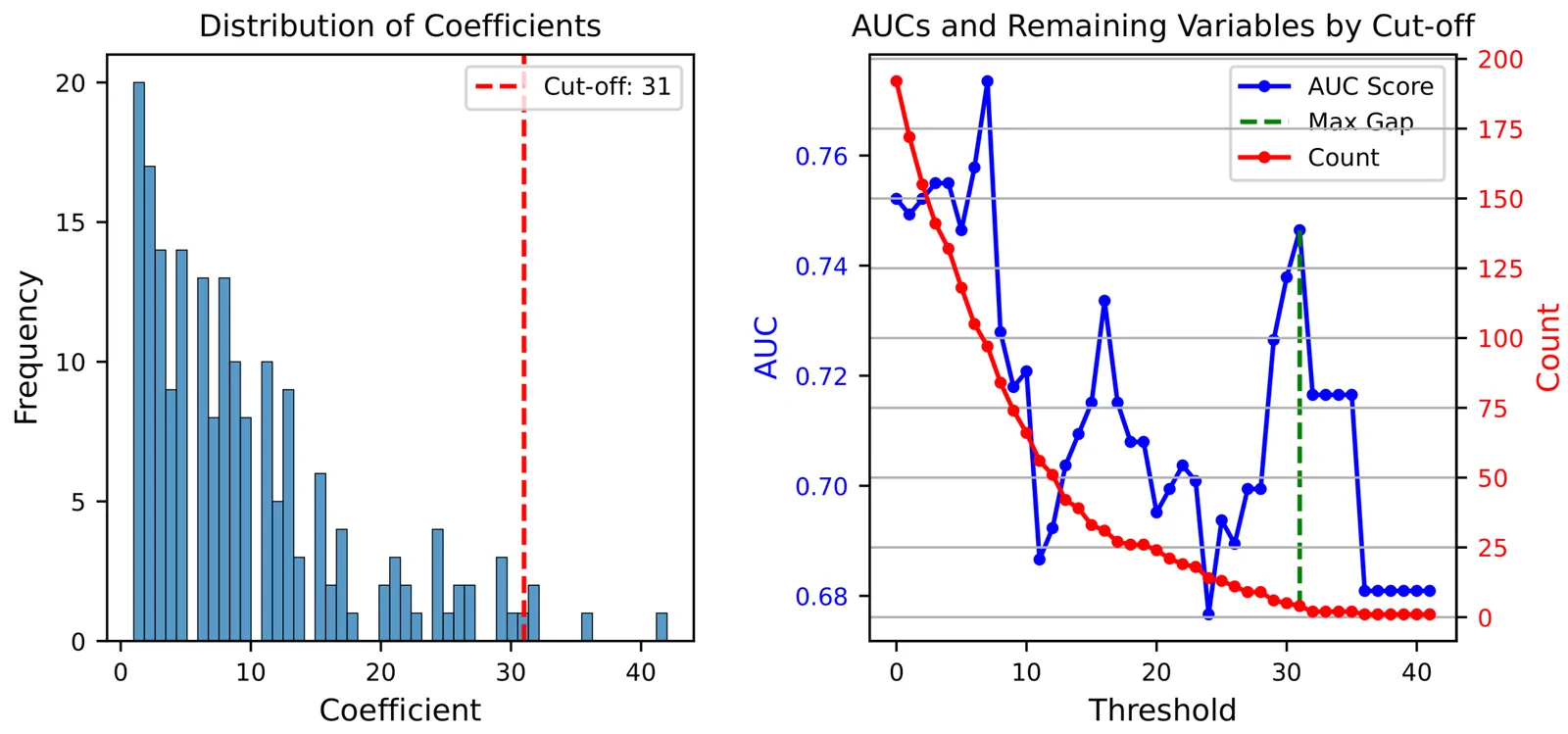
Distribution of SR coefficients and corresponding AUCs with variable counts across coefficient cut-offs.

After training, the SR model included 192 variables in the set *S*. However, the analysis of weight ( $w_{j}$ ) revealed that many variables ( $x_{j}$ ) had low contribution to the predictive performance of the model. Through the sequential reduction process, the optimal balance was observed at a cut-off of 31. This resulted in a reduced variable set size of S=4 while maintaining an AUC of 0.7464. As shown in Figure 8 and Table 5, the AUC even improved within certain cut-off ranges despite the reduction in model complexity, demonstrating an effective trade-off between set size and performance.

## 4. Discussion

This study compared traditional machine learning models and an SR-based approach for early prediction of BSI in liver transplant recipients. Overall, all models demonstrated modest discriminative performance (AUC 0.54–0.64), with no single model showing clearly superior performance. The SR model achieved comparable performance to conventional approaches, whereas the optimized SR model showed relatively lower performance.

These findings suggest that the SR model can provide interpretable risk equations to support clinical reasoning and decision-making. However, the overall predictive performance remains limited and may restrict its clinical applicability. The current level of discrimination may not be sufficient for reliable risk stratification at the individual patient level. This may lead to false-positive or false-negative predictions in clinical settings, resulting in unnecessary interventions and increased healthcare costs, or delayed diagnosis and treatment of true infections. Therefore, further improvements are required to enhance model performance, including refinement of feature selection, optimization of preprocessing strategies, and development of more advanced modeling approaches. Importantly, such improvements should aim to balance increased model complexity with the preservation of interpretability.

Analysis of feature importance consistently identified CRP, lymphocyte count, total bilirubin, creatinine, and sodium as key predictors, reflecting the well-recognized roles of systemic inflammation, hepatic dysfunction, renal impairment, and electrolyte imbalance in the development of BSIs. Notably, while Anti-HBs Ab was detected across multiple models (e.g., LR and SVM), the SR model placed specific emphasis on a more comprehensive viral panel, including anti-HBc Ab and anti-EBNA IgG (Figure 7). This pattern mirrors our Cox time-to-BSI analysis (Figure 3), in which HBV- and EBV-related markers were significantly associated with an increased hazard of post-transplant BSI. The convergence of evidence across these analytically distinct methods suggests that underlying viral immunologic status may play a meaningful role in shaping early post-transplant susceptibility to bacterial infection, extending beyond what is captured by standard laboratory indices alone. Prior studies suggest that latent herpesvirus infections, including EBV, can modulate host immune responses and may be associated with indirect effects such as increased susceptibility to other infections in solid-organ transplant recipients [55,56,57], whereas HBV-related serologic patterns—particularly anti-HBc positivity—identify individuals at risk of loss of immune control and HBV reactivation under immunosuppression [58,59]. More broadly, transplant infectious disease frameworks and guidelines emphasize that pretransplant viral serologic testing is central to infectious risk assessment and can shape early post-transplant infectious complications [60,61,62]. Although much of the consensus literature focuses on cytomegalovirus (CMV) as a prototypical example, the same conceptual framework—baseline viral immunity as a marker of immunologic vulnerability and a modifier of infectious risk—may also be relevant to other latent viruses in LT, such as EBV and HBV [60,61]. Taken together, the concordant identification of EBV/HBV-related serologies by SR and their association with time-to-BSI in the Cox model suggest that viral serologic profiles, often treated as background information, serve as practical indicators of immunologic vulnerability and should be considered in future risk-stratification models for post-transplant BSI [61,62].

Findings from Kaplan–Meier analysis showed that BSI occurred mainly in the early postoperative phase, with BSI-free survival declining steeply within the first 30 days (Figure 2). This early hazard window is consistent with the well-described post-transplant infection timeline. In this period, the first month after LT is dominated by nosocomial bacterial infections related to perioperative exposures and intensive immunosuppression [6,63,64]. This pattern underscores the importance of early prediction and focused monitoring, as timely intervention during this high-risk period may improve outcomes.

Notably, the SR model expressed risk factors as simple threshold-based rules, providing transparent predictive criteria. Consistent with SHAP analysis and direct inspection of the SR-derived rules (Figure 7 and Figure 8 and Table 4 and Table 5), the SR approach demonstrated a higher level of interpretability by explicitly specifying clinically meaningful cut-points and their directional contributions to risk. To facilitate interpretability, continuous laboratory variables were transformed into binary features based on clinical reference ranges. Although this may result in some loss of predictive information, it allows the resulting equations to be more intuitive and clinically interpretable. While alternative encoding strategies, such as multi-level discretization or nonlinear transformations, may improve predictive performance, they may also reduce interpretability. Therefore, such approaches should be carefully considered, with priority given to preserving interpretability.

The novelty of this study lies in the development of prediction models specifically tailored to liver transplant recipients and the introduction of interpretable risk equations using symbolic regression (SR) in conjunction with conventional machine learning models. However, the predictive performance in the held-out test set was modest, suggesting that preoperative (and limited perioperative) variables alone may be insufficient to reliably capture the multifactorial and dynamic mechanisms leading to early post-transplant BSI. In addition, for the SR model, the combination of extensive variable exploration with constraints imposed to ensure interpretability—specifically, binarization of variables and restriction of the mathematical expressions to the addition operator (‘+’)—may have contributed to the observed limitations in predictive performance. Additional limitations include the single-center retrospective design, a relatively small sample size, and the absence of external validation. These factors may limit the generalizability and stability of the proposed models, as model performance may vary across different clinical settings and patient populations. In particular, models developed from a single-center dataset may reflect institution-specific practices and patient characteristics, which can reduce their applicability to broader transplant populations. Therefore, external validation using independent multicenter cohorts is essential to confirm the robustness and generalizability of the proposed approach. Furthermore, the retrospective nature of the study limited the availability of detailed information on the origin of infection and the clonal relatedness of isolates, which may affect the interpretation of pathogen distribution. Future work should prioritize multicenter cohorts with external validation and consider incorporating time-varying postoperative signals (e.g., early laboratory trends and continuously monitored vital signs), perioperative antimicrobial exposure, and device-related factors to improve discrimination and calibration, thereby strengthening the generalizability and clinical utility of the proposed models. In addition, the retrospective nature of the study limited the availability of detailed information on the origin of infection and the clonal relatedness of isolates, which may affect the interpretation of pathogen distribution.

## 5. Conclusions

This study developed machine learning-based classification models to predict early BSI in liver transplant recipients and introduced an interpretable risk equation using SR. Overall, the models demonstrated modest discriminative performance (AUC 0.53–0.64), with comparable results across conventional machine learning approaches and the SR model.

These findings indicate that the current models have limited capability for reliable prediction at the individual patient level, highlighting the need for further improvement. The limited use of perioperative variables, along with the encoding strategy adopted to enhance interpretability, may have contributed to the observed performance.

Meanwhile, the results of SHAP-based interpretation and statistical analyses provided clinically meaningful insights into potential risk factors. Notably, viral serologic markers such as EBV- and HBV-related variables emerged as potential contributors, indicating that these markers may be associated with post-transplant BSI risk beyond conventional laboratory indicators.

Taken together, these findings suggest that interpretable modeling approaches can serve not only as predictive tools but also as a framework for identifying clinically relevant variables. Future studies should focus on improving model performance and generalizability while preserving interpretability, as enhanced predictive accuracy and robustness across diverse clinical settings may enable more reliable identification of clinically important risk factors and clearer assessment of their clinical impact.

## Figures and Tables

**Table 1:** Baseline characteristics and surgery-related variables. Numerical variables are expressed as mean (standard deviation), and categorical (qualitative) variables are presented as counts (n).

		BSI ^1^ (*n* = 82)	non-BSI (*n* = 163)	*p*-Value
Demographic				
Age		54.74 (10.86)	54.45 (10.03)	0.837
Weight		64.94 (9.55)	66.35 (12.43)	0.325
Height		165.29 (7.98)	166.64 (8.02)	0.214
Sex	Male	55	121	0.292
	Female	27	42	
Missing weight	False	63	139	0.111
	True	19	24	
Missing height	False	68	148	0.093
	True	14	15	
Clinical status before operation				
LOC ^2^	alert	45	130	<0.001 ***
	drowsy	13	16	
	stupor	3	4	
	sedation	21	13	
Ward type	ICU ^3^	45	53	0.001 ***
	general	37	110	
Ventilation	False	59	146	0.001 ***
	True	23	17	
CRRT ^4^	False	39	114	0.001 ***
	True	43	49	
Surgery				
Transfusion	RBC ^5^	9.88 (5.42)	7.68 (6.34)	0.005 **
	PLT ^6^	1.57 (1.87)	1.54 (3.13)	0.917
	FFP ^7^	9.91 (5.58)	7.77 (6.66)	0.009 **
LT type	DDLT ^8^	51	75	0.021 *
	LDLT ^9^	31	88	

^1^ BSI: bloodstream infection group, ^2^ LOC: Level of consciousness ^3^ ICU: Intensive care unit ^4^ CRRT: Continuous renal replacement therapy ^5^ RBC: Red blood cell ^6^ PLT: Platelet ^7^ FFP: Fresh frozen plasma ^8^ DDLT: Deceased donor liver transplantation ^9^ LDLT: Living donor liver transplantation. * *p* < 0.05, ** *p* < 0.01, *** *p* < 0.001.

**Table 2:** Distribution of causative pathogens in BSI.

Bacteria	Count
*Enterococcus faecium*	17
*Staphylococcus aureus*	11
*Klebsiella pneumoniae*	10
*Escherichia coli*	8
*Enterococcus faecalis*	6
*Acinetobacter baumannii*	6
*Candida glabrata*	5
*Staphylococcus epidermidis*	3
*Streptococcus mitis/oralis*	3
*Pseudomonas aeruginosa*	2
*Actinomyces odontolyticus*	2
*Corynebacterium striatum*	2
*Candida tropicalis*	2
*Candida albicans*	2
*Candida pelliculosa*	1
*Parabacteroides merdae*	1
*Enterobacter cloacae/asburiae*	1
*Gram (+) nonsporeforming bacilli*	1
*Raoultella ornithinolytica*	1
*Staphylococcus haemolyticus*	1
*Stenotrophomonas maltophilia*	1
*Unidentified Yeast*	1

**Table 3:** Model performance metrics with 95% confidence intervals.

Model	AUC	Accuracy	Recall	F1-Score
Support vector machine	0.59 (0.41–0.77)	0.47 (0.33–0.61)	**0.76** (0.55–0.95)	0.50 (0.32–0.65)
Random forest	**0.64** (0.47–0.81)	0.59 (0.45–0.73)	0.65 (0.40–0.87)	**0.52** (0.32–0.69)
Logistic regression	0.54 (0.35–0.72)	**0.61** (0.47–0.76)	0.47 (0.25–0.71)	0.46 (0.25–0.65)
XGBoost	0.58 (0.40–0.75)	0.57 (0.43–0.71)	0.65 (0.42–0.88)	0.51 (0.32–0.68)
Symbolic regression	0.63 (0.46–0.80)	0.57 (0.45–0.69)	0.65 (0.40–0.85)	0.51 (0.31–0.68)
Symbolic regression_optimized	0.55 (0.38–0.73)	0.55 (0.41–0.69)	0.59 (0.33–0.82)	0.48 (0.26–0.64)

Bold values indicate the highest performance for each evaluation metric.

**Table 4:** Range and score table of the top 10 terms.

Variable	Range	Score
RBC transfusion	≥12.0	57
LOC_preop	≥Stupor	44
AntiHBc Ab_preop	<PosGray	44
AntiHbe Ab_preop	<NegGray	43
CRRT_preop	==True	42
Max OR Temp	<36.0	41
RBC transfusion	≥4.0 and <12.0	41
Cr_adm	≥1.2	40
BUN_adm	≥23.6	38
Max OR Temp	<36.0	38

**Table 5:** Range and score table of optimized model terms.

Variable	Range	Score
AntiHBc Ab_preop	<PosGray	32
MaxORTemp	<36.5	36
CRRT_preop	==True	42
Cr_adm	≥0.7	32

## Data Availability

The data underlying this article cannot be shared publicly because of patient privacy and ethical restrictions. The data will be shared on reasonable request to the corresponding authors.

## Abbreviations

The following abbreviations are used in this manuscript:

Ab	Antibody
Ag	Antigen
ALP	Alkaline Phosphatase
ALT	Alanine Aminotransferase
AUC	Area Under the Curve
AST	Aspartate Aminotransferase
BSI	Bloodstream Infection
BUN	Blood Urea Nitrogen
CKD	Chronic Kidney Disease
CLD	Chronic Lung Disease
CMV	Cytomegalovirus
CRP	C-reactive Protein
CRRT	Continuous Renal Replacement Therapy
Cr	Creatinine
DB	Direct Bilirubin
DDLT	Deceased Donor Liver Transplantation
DM	Diabetes Mellitus
EBNA	Epstein–Barr Nuclear Antigen
EBV	Epstein–Barr Virus
EMR	Electronic Medical Record
FFP	Fresh Frozen Plasma
HCC	Hepatocellular Carcinoma
HCV	Hepatitis C Virus
HE	Hepatic Encephalopathy
HIV	Human Immunodeficiency Virus
HBV	Hepatitis B Virus
HTN	Hypertension
HR	Hazard Ratio
ICU	Intensive Care Unit
IgG	Immunoglobulin G
IgM	Immunoglobulin M
KM	Kaplan–Meier
LDLT	Living Donor Liver Transplantation
LT	Liver Transplantation
LOC	Level of Consciousness
LR	Logistic Regression
LR-L1	L1-Regularized Logistic Regression
ML	Machine Learning
Na	Sodium
ND	Not Detected
OR	Operating Room
PCR	Polymerase Chain Reaction
PLT	Platelet
PT-INR	Prothrombin Time–International Normalized Ratio
RBC	Red Blood Cell
ReTx	Re-transplantation
RF	Random Forest
ROC-AUC	Receiver Operating Characteristic–Area Under the Curve
SHAP	Shapley Additive Explanations
SR	Symbolic Regression
SVM	Support Vector Machine
TB	Total Bilirubin
VCA	Viral Capsid Antigen
WBC	White Blood Cell
XGBoost	Extreme Gradient Boosting

## Appendix A

**Table A1:** Medical history. Variables are presented as counts.

		BSI ^1^ (*n* = 82)	Non-BSI (*n* = 163)	*p*-Value
Medical History				
CLD ^2^	False	82	162	1.000
	True	0	1	
HTN ^3^	False	72	142	1.000
	True	10	21	
Medical History				
DM ^4^	False	63	122	0.875
	True	19	41	
HCC ^5^	False	61	112	0.377
	True	21	51	
HE ^6^	False	54	122	0.175
	True	28	41	
CKD ^7^	False	79	158	1.000
	True	3	5	

^1^ BSI: bloodstream infection group, ^2^ CLD: Chronic lung disease, ^3^ HTN: Hypertension, ^4^ DM: Diabetes mellitus, ^5^ HCC: Hepatocellular carcinoma, ^6^ HE: Hepatic encephalopathy, ^7^ CKD: Chronic kidney disease.

**Table A2:** Clinical status on admission. Variables are presented as counts.

		BSI (*n* = 82)	Non-BSI (*n* = 163)	*p*-Value
Clinical status on admission				
LOC ^1^	alert	69	145	0.507
	drowsy	10	15	
	stupor	3	3	
Ward type	ICU ^2^	40	52	0.012 *
	general	42	111	
Ventilation	False	80	158	1.000
	True	2	5	
CRRT ^3^	False	74	153	0.310
	True	8	10	

^1^ LOC: Level of consciousness, ^2^ ICU: Intensive care unit, ^3^ CRRT: Continuous renal replacement therapy. * *p* < 0.05.

**Table A3:** Laboratory test results. Values are mean (standard deviation).

		BSI (*n* = 82)	Non-BSI (*n* = 163)	*p*-Value
On admission	WBC ^1^	9.78 (7.13)	8.28 (7.01)	0.118
	PLT ^2^	90.44 (58.38)	93.52 (65.56)	0.709
	CRP ^3^	2.47 (2.53)	2.50 (2.29)	0.928
	TB ^4^	18.40 (14.90)	12.29 (12.97)	0.002 **
	DB ^5^	7.09 (8.47)	5.05 (7.22)	0.064
	AST ^6^	227.66 (441.18)	122.82 (268.34)	0.051
	ALT ^7^	351.66 (1841.29)	107.15 (555.75)	0.243
	ALP ^8^	171.59 (176.73)	153.65 (106.06)	0.400
	BUN ^9^	36.12 (25.92)	27.28 (22.47)	0.009 **
	Cr ^10^	2.17 (2.07)	1.56 (1.65)	0.021 *
	PT-INR ^11^	2.83 (4.11)	1.97 (1.20)	0.067
Preoperation	WBC	9.17 (6.14)	7.32 (7.03)	0.036 *
	PLT	65.40 (40.86)	76.16 (53.32)	0.082
	CRP	3.20 (3.89)	2.36 (1.97)	0.069
	TB	20.62 (15.68)	13.84 (15.23)	0.002 **
	AST	240.71 (1134.95)	84.15 (98.71)	0.216
	ALT	126.95 (580.67)	64.02 (269.76)	0.354
	ALP	143.78 (148.67)	136.77 (92.37)	0.697
	BUN	25.93 (17.84)	22.13 (21.25)	0.142
	Cr	1.46 (1.01)	1.32 (1.39)	0.352
	PT-INR	2.53 (1.53)	1.93 (0.85)	0.001 **

^1^ WBC: White blood cell, ^2^ PLT: Platelet, ^3^ CRP: C-reactive protein, ^4^ TB: Total bilirubin, ^5^ DB: Direct bilirubin, ^6^ AST: Aspartate aminotransferase, ^7^ ALT: Alanine aminotransferase, ^8^ ALP: Alkaline phosphatase, ^9^ BUN: Blood urea nitrogen, ^10^ Cr: Creatinine, ^11^ PT-INR: Prothrombin time–International normalized ratio. * *p* < 0.05, ** *p* < 0.01.

**Table A4:** Baseline infectious disease test of patients groups. Numerical variables are expressed as mean (standard deviation), and categorical (qualitative) variables are presented as counts (n).

			BSI (*n* = 82)	Non-BSI (*n* = 163)	*p*-Value
Infectious Disease Test					
CMV ^1^	PCR ^2^		36.94 (206.05)	7.58 (38.91)	0.205
	IgG ^3^		69.03 (42.74)	70.96 (47.61)	0.748
	IgM ^4^		0.03 (0.24)	1.06 (12.84)	0.308
EBV ^5^	PCR		0.00 (0.00)	13.80 (130.89)	0.180
	antiEBNA ^6^ IgG		4.18 (4.37)	5.23 (4.29)	0.076
	antiEBNA IgM		4.79 (15.68)	2.82 (6.73)	0.279
	VCA ^7^ IgG		3.74 (2.00)	3.76 (2.05)	0.939
	VCA IgM		0.01 (0.03)	0.08 (0.70)	0.189
HBV ^8^	PCR		47,846.10 (294,974.07)	9649.65 (83,161.81)	0.253
	AntiHBsAb ^9^		95.22 (241.97)	111.59 (237.47)	0.616
	HBe Ag ^10^		15.44 (101.75)	16.02 (96.96)	0.966
	HBs Ag ^11^	negative	60	107	0.248
		positive	22	56	
	AntiHBe Ab ^12^	negative	62	118	0.528
		negative gray	0	4	
		positive gray	1	3	
		positive	19	38	
	AntiHBc Ab ^13^	negative	40	71	0.715
		negative gray	1	4	
		positive gray	0	1	
		positive	41	87	
HCV ^14^	PCR		0.37 (2.33)	26,170.21 (227,154.25)	0.143
	HCV Ab	negative	80	153	0.347
		positive	2	10	
HIV ^15^	HIV Ab	negative	82	162	1.000
		positive	0	1	

^1^ CMV: Cytomegalovirus, ^2^ PCR: Polymerase chain reaction, ^3,4^ IgG/IgM: Immunoglobulin G/M, ^5^ EBV: Epstein–Barr virus, ^6^ antiEBNA: Anti–Epstein–Barr nuclear antigen, ^7^ VCA: Viral capsid antigen, ^8^ HBV: Hepatitis B virus, ^9^ AntiHBs Ab: Antibody to hepatitis B surface antigen, ^10^ HBe Ag: Hepatitis B e antigen, ^11^ HBs Ag: Hepatitis B surface antigen, ^12^ AntiHBe Ab: Antibody to hepatitis B e antigen, ^13^ AntiHBc Ab: Antibody to hepatitis B core antigen, ^14^ HCV: Hepatitis C virus, ^15^ HIV: Human immunodeficiency virus.

**Table A5:** Other surgery variables. Numerical variables are expressed as mean (standard deviation), and categorical variables are presented as counts (n).

		BSI (*n* = 82)	Non-BSI (*n* = 163)	*p*-Value
Surgery				
OR Temp ^1^	min	34.39 (0.92)	34.51 (0.94)	0.313
	max	36.19 (0.73)	36.33 (0.66)	0.130
ReTx ^2^	False	78	159	0.447
	True	4	4	

^1^ OR Temp: Minimum/Maximum body temperature during operation, ^2^ ReTx: Re-transplantation.

**Table A6:** Blood culture contaminants.

Organism
*Staphylococcus warneri*
*Staphylococcus hominis*
*Staphylococcus capitis*
*Staphylococcus cohnii* ssp. *cohnii*
*Staphylococcus pettenkoferi*
*Staphylococcus epidermidis*
*Micrococcus luteus/lylae*
*Aerococcus viridans*
*Corynebacterium striatum*
*Bacillus cereus*
*Brevibacillus* spp.
*Paenibacillus provencensis*
*Gram-positive spore-forming bacilli*
*Gram-positive non-spore-forming bacilli*
*Unidentified Gram-positive cocci*
